# Burnout Among Psychiatry Doctors in Pakistan: A Multicenter Study

**DOI:** 10.1192/j.eurpsy.2025.1622

**Published:** 2025-08-26

**Authors:** G. Murtaza

**Affiliations:** 1 Psychiatry, Jannat Medical Complex Rawalpindi, Rawalpindi; 2 Psychiatry, Armed Forces Institute of Mental Health Rawalpindi, Rawalpindi, Pakistan; 3 Psychiatry, North Dublin Mental Health Services, Dublin, Ireland

## Abstract

**Introduction:**

Burnout among medical professionals, particularly psychiatry doctors, is a pressing concern. High-stress environments, heavy workloads, and emotional demands can lead to physical, emotional, and mental exhaustion. Pakistani psychiatry trainees face unique challenges, including intensive training, high patient volumes, and limited support. Despite its implications for patient care and doctor well-being, limited research exists on burnout among Pakistani psychiatry doctors.

**Objectives:**

To investigate burnout prevalence, contributing factors, effects, and coping strategies among Pakistani psychiatry trainees, informing evidence-based interventions to promote well-being and improve patient care.

**Methods:**

A cross-sectional online survey was conducted among psychiatry doctors from 10 hospitals in Pakistan (July 26, 2024 – September 25, 2024). The questionnaire assessed demographic characteristics, burnout factors, effects, and coping strategies.

**Results:**

The survey revealed significant burnout factors, including insufficient support from colleagues/administration (42.3%), high workload (23.1%), personal life stressors (19.2%), long working hours (11.5%), and lack of control (3.5%). Burnout effects included physical health issues (30.8%), mental health issues (30.8%), relationship strain (25.4%), and reduced job satisfaction (23.1%). Participants employed various coping strategies, such as having fun/leisure activities (38.5%), time management techniques (29.2%), engaging in self-care activities (26.9%), setting boundaries at work (11.5%), and seeking professional help (3.9%). Notably, the majority of participants (80.8%) were trainees, highlighting the vulnerability of this group to burnout.

**Conclusions:**

This study highlights the alarming prevalence of burnout among Pakistani psychiatry doctors, underscoring the need for targeted interventions to promote support, workload management, and work-life balance.

**Disclosure of Interest:**

None Declared

